# Elevated air humidity affects hydraulic traits and tree size but not biomass allocation in young silver birches (*Betula pendula*)

**DOI:** 10.3389/fpls.2015.00860

**Published:** 2015-10-13

**Authors:** Arne Sellin, Katrin Rosenvald, Eele Õunapuu-Pikas, Arvo Tullus, Ivika Ostonen, Krista Lõhmus

**Affiliations:** Institute of Ecology and Earth Sciences, University of TartuTartu, Estonia

**Keywords:** biomass allocation, climate change, Huber value, hydraulic architecture, hydraulic conductance, wood density

## Abstract

As changes in air temperature, precipitation, and air humidity are expected in the coming decades, studies on the impact of these environmental shifts on plant growth and functioning are of major importance. Greatly understudied aspects of climate change include consequences of increasing air humidity on forest ecosystems, predicted for high latitudes. The main objective of this study was to find a link between hydraulic acclimation and shifts in trees’ resource allocation in silver birch (*Betula pendula* Roth) in response to elevated air relative humidity (RH). A second question was whether the changes in hydraulic architecture depend on tree size. Two years of application of increased RH decreased the biomass accumulation in birch saplings, but the biomass partitioning among aboveground parts (leaves, branches, and stems) remained unaffected. Increased stem Huber values (xylem cross-sectional area to leaf area ratio) observed in trees under elevated RH did not entail changes in the ratio of non-photosynthetic to photosynthetic tissues. The reduction of stem–wood density is attributable to diminished mechanical load imposed on the stem, since humidified trees had relatively shorter crowns. Growing under higher RH caused hydraulic conductance of the root system (*K*_R_) to increase, while *K*_R_ (expressed per unit leaf area) decreased and leaf hydraulic conductance increased with tree size. Saplings of silver birch acclimate to increasing air humidity by adjusting plant morphology (live crown length, slenderness, specific leaf area, and fine-root traits) and wood density rather than biomass distribution among aboveground organs. The treatment had a significant effect on several hydraulic properties of the trees, while the shifts were largely associated with changes in tree size but not in biomass allocation.

## Introduction

For Europe, climate change is predicted to bring about both a decrease (southern and central Europe) as well as increase (northern Europe) in precipitation and environmental humidity (IPCC, 2013). The shortage of water reduces tree growth because of suppression of their photosynthesis, restricted stomatal conductance to avoid water loss through transpiration, and impeded nutrient uptake (Hsiao, 1973; Flexas and Medrano, 2002). Many studies have proven that drought significantly reduces tree diameter increment across mid- and southern-Europe (Pasho et al., 2012; Weemstra et al., 2013; Lévesque et al., 2014), and even low air relative humidity (RH) alone results in declined productivity and biomass of European beech (Lendzion and Leuschner, 2008). The forest decline attributable to climate warming and increased frequency of weather extremes is associated with hydraulically mediated carbon starvation and subsequent predisposition to attack from biotic agents (McDowell et al., 2008; Choat et al., 2012; Nardini et al., 2013). The knowledge of the influence of increased precipitation or air humidity on tree growth allocation and the concurrent hydraulic acclimation is much more limited. Increase in mean annual precipitation is strongly correlated with precipitation frequency (Räisänen et al., 2004), whereas rising rainfall frequency inevitably results in higher mean RH at local or regional scales. Increasing air humidity commonly reduces water fluxes through plants (Fanourakis et al., 2011; Kupper et al., 2011; Hu et al., 2012), and, as a consequence, the uptake and mass flow of soluble minerals are impeded (Cramer et al., 2009; Tullus et al., 2012), as in the case of drought. Diminished nutrient uptake from the soil in turn decreases leaf nutritional status causing lower photosynthetic capacity and growth rate (Sellin et al., 2013). Nevertheless, in northern Europe, tree-ring width and summer precipitation are not clearly related (Babst et al., 2013). Therefore, more experimental research in tree carbon allocation and storage under environmental stress in relation to global change is needed (Niinemets, 2010).

Biomass accumulation unequivocally expresses the efficiency of a tree’s performance in given growth conditions, since most stress factors impede plant net assimilation. Biomass distribution between functionally most active tree parts (leaves and fine roots) can indicate where (above or below ground) the most growth-limiting resource is located. This assumption is based on the multiple limitation hypothesis: plants adjust the growth of different organs so that all essential resources (light, CO_2_, water, and mineral nutrients) limit equally (Bloom et al., 1985). Biomass allocation among and within plant organs is an important feature interrelated with plant hydraulic properties (Magnani et al., 2000; Davi et al., 2009; Domec et al., 2012). The hydraulic architecture of a tree determines the ability to transfer water from soil to leaves, thus affecting its photosynthetic capacity, nutrient supply, and, therefore, the tree growth. Greater water transport (sap flow) or efficiency of the transport (greater hydraulic conductance at a given water potential gradient) should support higher carbon gain and growth rates (Smith and Sperry, 2014). Biomass production and water exchange between the canopy and atmosphere are both affected by leaf biomass and area, making them important plant functional traits (Poorter et al., 2012).

A previous study at the Free Air Humidity Manipulation (FAHM) site revealed that increased air humidity induced some changes in hydraulic properties of silver birch (*Betula pendula*) saplings (Sellin et al., 2013). Huber values (HV), defined as the ratio of stem xylem cross-sectional area to leaf area supported by the respective stem segment, increased, leading to higher leaf-specific conductivity of stems. Also, hydraulic conductance of the root system increased, while hydraulic conductance of leaf blades and stem–wood density exhibited a decreasing trend. Increase of HV seems to be more a common response to elevated RH observed both in saplings of silver birch and coppice of hybrid aspen. Larger HV in trees grown under higher air humidity implies larger resource allocation to the water-conducting system relative to the foliage area. Larger investments in xylem result in an increase in the ratio of non-photosynthetic to photosynthetic tissues and in larger maintenance respiration costs determined by the volume of living parenchyma cells (Ryan, 1990; Carey et al., 1997; Maier et al., 1998). The shift in resource allocation may contribute to the growth deceleration of high-humidity grown trees, recorded in both tree species in the FAHM experiment (Tullus et al., 2012; Sellin et al., 2013).

Our understanding of how high humidity affects stomatal morphology and regulation is still scanty and experimental data are controversial. Leaves grown in high RH have bigger stomata, higher variability in stomatal size, and exhibit usually lower stomatal sensitivity to closing stimuli than in plants grown under moderate RH (Arve et al., 2013; Aliniaeifard et al., 2014). Recent results obtained from the FAHM experiment revealed opposite responses: stomata of both silver birch (Sellin et al., 2014) and hybrid aspen (Niglas et al., 2015) developed in elevated air humidity are more sensitive to changes in environmental factors. Thus, further studies on stomatal regulation, co-ordination of stomatal and hydraulic conductances and their role in driving biomass allocation are necessary.

Sellin et al. (2013) presented data on the impact of elevated air humidity on hydraulic properties of silver birch based on measurements of separate sample branches or stem segments taken from three canopy positions. The current paper aims to find whether the shifts in birch saplings hydraulic parameters (averaged across the whole crown) and nutrient status, caused by humidification, bring about changes in resource allocation pattern as well as biomass accumulation. In addition, we address a question whether the changes observed in hydraulic architecture in response to increased RH depend on tree size. We hypothesize: (1) elevated air humidity increases the ratio of non-photosynthetic to photosynthetic tissues, reflected by changes in HVs; (2) the reduction of wood density under high RH-grown trees is associated with biomass distribution among stem, branches, and leaves; and (3) the elevated humidity-driven changes depend on tree size. Determining the effects of increasing air humidity on biomass allocation and accumulation of silver birch and linking them with changes in trees’ hydraulic properties should help to disclose the mechanisms behind the acclimation processes of deciduous tree species to climate trends predicted for high latitudes.

## Materials and Methods

### Study Area and Sample Trees

The studies were performed on silver birch (*B. pendula* Roth) in an experimental forest plantation at the FAHM site, situated at Rõka village (58°14′ N, 27°17′ E, 40–48 m above sea level), south-eastern Estonia. The long-term average annual precipitation in the region is 650 mm, and the average temperature is 17.0°C in July and -6.7°C in January. The growing season usually lasts 175–180 days from mid-April to October. The soil is a fertile Endogenic Mollic Planosol (WRB) with an A-horizon thickness of 27 cm. Total nitrogen (N) content in the A horizon is 0.11–0.14%, the C/N ratio is 11.4, and the pH is 5.7–6.3.

The study site, established on an abandoned agricultural field in 2006–2007, is a fenced area of 2.7 ha consisting of nine experimental plots (∅ 14 m) planted with silver birch and hybrid aspen (*Populus tremula* L. × *P. tremuloides* Michx.) and surrounded by a buffer zone. One-year-old seedlings of silver birch were planted in the experimental area in spring of 2006. The stand density in the buffer zone is 2,500 trees ha^-1^ and, in the experimental plots, 10,000 trees ha^-1^. Three sample plots were used as control (**C**) areas, and three plots were humidified (**H**). The computer-operated FAHM system, based on an approach integrating two different technologies (a misting technique to atomize/vaporize water and a FACE-like technology to mix humidified air inside the plots), enables the RH of the air to increase by up to 18% over the ambient level during humidification treatment, depending on the wind speed inside the experimental stand. The humidification was applied 6 days a week, during the daytime if ambient RH was <75% and mean wind speed <4 m s^-1^. As a long-term average, RH was increased by 7–8%. A detailed description of the FAHM site and technical setup is presented in Kupper et al. (2011). The treatment was started in June of 2008. Tree height (*h*) and stem diameter at a 30-cm height (*d*) of all trees in experimental plots were measured after the end of growing periods of 2008 and 2009 (**Table 1**). Stem volume index (*d^2^h)* was calculated as stem diameter squared multiplied by stem height. The ratio between tree height and diameter was defined as stem slenderness. Data on tree height, stem diameter, and slenderness have been published in Sellin et al. (2013). Six model trees per treatment (two trees per sample plot) were sampled for biomass distribution and hydraulic conductance evaluation from the end of July to the beginning of August in 2009. The height of the model trees varied between 3.0 and 3.5 m.

**Table 1:** Stand characteristics measured in 2009.

	C	H
Stem volume index, *d^2^h* (cm^3^)	4643 ± 253	3741 ± 210^∗∗^
Basal area (cm^2^)	9.54 ± 0.42	8.96 ± 0.40
Relative height increment (%)	46.5 ± 1.02	34.0 ± 1.43
Relative diameter increment (%)	54.1 ± 1.03	47.3 ± 1.54^∗∗∗^
Relative stem volume increment (%)	252 ± 6.0	195 ± 7.7^∗^
Relative basal area increment (%)	139 ± 3.2	122 ± 4.6^∗∗∗^

*Relative annual increment of a growth characteristic (X) was calculated as 100 × (X_2009_ – X_2008_)/X_2008_. The significant effect of humidification on stand characteristics (mixed model) is indicated by asterisks (^∗^P < 0.05, ^∗∗^P < 0.01, ^∗∗∗^P < 0.001).*

### Aboveground Biomass Distribution

The stems of model trees were divided into four sections: the stem up to the living crown, and the living crown divided into three sections. The fresh mass of each section was determined. Every living crown section was divided into fractions of stem, branches, and leaves. From every fraction, a subsample was taken for estimation of dry matter content as well as for chemical analysis. The subsamples were dried at 70°C until constant weight and weighed to 0.01 g. The dry masses of leaf (*M*_*fol*_), branch, and stem fractions were calculated for each model tree by multiplying the corresponding fresh mass by the dry matter ratio.

Wood densities of stem sections and model branches were estimated by dividing the dry mass of a specimen by its fresh volume (calculated from dimensions of the specimen approximated by a frustum of elliptical cone). The mean stem–wood density of a tree was calculated as the weighted average of specimen densities (weighted by the leaf area supported by respective stem sections). The share of the wood and bark of the stem were determined using disks cut from the middle of three lower stem sections.

To estimate the aboveground biomass and its fractions for all trees in experimental plots, regression models were developed based on the data of model trees (**Table 2**). The statistical model was a linear regression model

**Table 2:** Regression equations for estimating biomass of aboveground tree compartments (g) and tree leaf area (cm^2^), where *d*^2^*h* = stem volume index (cm^3^), *R*^2^ = coefficient of determination, and *P* = level of probability.

Regression equations		*R^2^*	*P*	SEE	RSE (%)
Aboveground biomass	=0.154 × *d^2^h*+ 287	0.61	<0.01	170.4	16
Stem biomass	=0.0899 × *d^2^h* + *101*	0.96	<0.001	27.3	5
Stem + Branch biomass	=0.120 × *d^2^h* + 241	0.62	<0.01	129	15
Foliar biomass	=0.0337 × *d^2^h* + 45.6	0.39	<0.05	57.6	27
Tree foliage area	=0.0007 × *d^2^h*+ 0.514	0.53	<0.01	8962	22

*N = 6 trees per treatment. Statistically insignificant intercepts are indicated in italics, SEE, standard error of estimate; RSE, relative standard error of estimate.*

(1) y=ax+b

where *x* is stem volume index (*d^2^h*). The treatment effect was not significant in the models.

### Fine Root Biomass

Fine-root (∅ < 2 mm) biomass of birch saplings was estimated in the A horizon (up to the sandy loam of the subsoil) using soil cores. Eighteen soil cores (∅ 48 mm) per treatment (six cores per plot) were taken at the beginning of July and separated into 10-cm layers. The roots of a soil core layer were carefully washed clean of the soil particles, and living fine roots were separated under a microscope from dead and coarse roots of birch. The birch fine-root biomass samples were dried at 70°C for 48 h and weighed to 0.001 g.

### Leaf Area and Chemical Characteristics

The leaf-blade area of all leaves of the sample branches was measured with an LI-3100C optical area meter (LI-COR Biosciences, Lincoln, NE, USA). To calculate a tree’s total foliage area, a regression model was used (**Table 2**). Leaf area ratio (LAR) was considered as the total leaf area per unit tree aboveground dry mass.

Leaf N concentrations were determined by block digestion and steam distillation methods (Kjeltec Auto 1030 Analyzer, FOSS Tecator AB, Höganäs, Sweden). Phosphorus (P) concentrations of plant material were determined spectrophotometrically from Kjeldahl digests using a FIAstar 5000 Analyzer (FOSS Tecator AB). Chemical analyses were performed at the Laboratory of Biochemistry, Estonian University of Life Sciences.

### Hydraulic Measurements

Three branches and three stem segments from each sample tree were measured for hydraulic properties. The sample branches were cut from three heights in the canopy: on average at 53 (mean length 91 cm), 177 (mean length 112 cm), and 227 cm (mean length 91 cm) above the ground. The stem specimens (15–20 cm long) were cut on average from heights of 264, 158, and 25 cm above the ground. Hydraulic conductance of whole branches (*K*_sh_) and their parts - leafless branch (*K*_B_) and leaves (*K*_L_) - was determined by the water perfusion method using a high pressure flow meter (HPFM; Dynamax, Houston, TX, USA) applied in a quasi-steady-state mode. All leaves of the sample branches were collected and the total area of leaf blades was measured with a LI-3100C. Immediately after sampling of the lowest stem segment for specific hydraulic conductivity of branch-wood (*k*_B_), the absolute hydraulic conductance of the root system (*K*_Rabs_) was measured *in situ* with the HPFM applied in a transient mode. The whole procedure has been described in detail in Sellin et al. (2013). Contribution of bare branch to branch total hydraulic resistance (*R*_B_) was quantified as follows:

(2) $$R_{B}=\frac{K_{B}^{-1}}{K_{sh}^{-1}}\,$$

Analogically was calculated the contribution of leaves to branch total hydraulic resistance, i.e., relative leaf hydraulic resistance (*R*_L_). Total foliage hydraulic conductance (*K*_fol_) was calculated by multiplying foliage area (*A*_fol_) by mean *K*_L_ weighted by leaf area of the sample branches. Huber values (HV) were calculated as the cross-sectional area of stems or branches divided by foliage area supported by the corresponding stem or branch sections. Tree-level means of hydraulic parameters were calculated as averages weighted by respective foliage areas located distally of the sampling points.

### Data Analysis

Statistical data analysis was carried out using Statistica, version 7.1 (StatSoft, Inc., Tulsa, OK, USA) software package. Unless otherwise noted, the level of significance α = 0.05 was accepted. The effect of humidification on plant functional traits (expressed as tree-level means weighted by respective foliage areas located distally of the sampling point) was assessed with a following mixed model:

(3) $$y_{ijk}=\mu +\beta_{i}+b_{j}+\varepsilon_{ijk}$$

where *y*_ijk_ is the studied characteristic of the kth sample tree from plot j in treatment *i*, μ is the grand mean, *β_i_* is the fixed effect of treatment (control or humidification), *b_j_* is the random effect due to jth plot [*b_j_* ∼*N*(0; $\sigma_{b}^{2}$)], and 𝜀_ijk_ is random residual error [𝜀_ijk_ ∼*N*(0; σ^2^)]. When analyzing leaf data sampled from three different heights from each tree, sample tree was included as another random factor, and relative height (continuous covariate) and its interaction with treatment were included as fixed factors. Type III sums of squares with Satterthwaite approximation for degrees of freedom were used in the calculations. For testing possible tree-size-related effects on hydraulic parameters, different size parameters (total foliage area, aboveground biomass, tree height, etc.) were included in the analyses. Generalized linear/non-linear model (GLZ) analysis was used for detecting the influence of humidification on allometric regression models. The significant categorical factors and predictors were determined using Type-3 LR tests. Bivariate relationships between the studied characteristics and independent variables were assessed by the Pearson correlation coefficient and by simple linear or non-linear regression procedures based on the least-squares method. If humidification significantly impacted a relationship (GLZ), separate correlation coefficients for treatments were calculated.

## Results

### Biomass of Young Birches

Two years of humidification did not significantly affect the biomass allocation among foliage, branches, and stems in birch saplings (**Table 3**). However, the stem volume index (*d*^2^*h*) and most relative increments of tree size parameters were significantly smaller in **H** plots (**Table 1**). Humidification decreased biomasses of all the measured compartments (stems, branches, leaves, and fine roots), and the aboveground biomass remained 14% lower than that in **C** stands (**Table 4**). In addition to decreased stand aboveground biomass and tree size, humidification reduced mean stem–wood density as well as the live crown ratio of trees (**Table 3**). Stem–wood density increased with foliar biomass (*R* = 0.63, *P* = 0.028) and live crown ratio (*R* = 0.64, *P* = 0.024). The stem slenderness was negatively correlated with branch proportion in aboveground biomass (*R* = -0.73, *P* < 0.01). In addition, a significant correlation appeared between mean branch-wood density (Sellin et al., 2013) and leaf proportion in aboveground biomass (*R* = 0.76, *P* < 0.01). The ratios between stem- and foliage-size parameters did not change significantly as a result of humidification treatment (**Table 3**). Biomass distribution among the aboveground parts of trees did not depend on tree size.

**Table 3:** Biomass allocation, wood density, and leaf area ratio (mean ± SE) of birch saplings in control (**C**) and humidified plots (**H**).

	C	H	
Stem %	54.4 ± 1.3	51.4 ± 3.4	
Branches %	24.8 ± 1.0	29.1 ± 3.3	
Leaves %	20.8 ± 0.8	19.5 ± 2.1	
Bark % in stem biomass	22.7 ± 1.4	22.7 ± 1.3	
Stem wood density (g cm^-3^)	0.381 ± 0.007	0.361 ± 0.008^∗^
Live crown ratio	0.900 ± 0.007	0.868 ± 0.010^∗^
*M*_stem_:*M*_fol_	2.63 ± 0.14	2.85 ± 0.47	
*d^2^h:M*_fol_ (cm^3^ g^-1^)	24.5 ± 1.7	25.1 ± 3.9	
*d^2^h:A*_fol_ (cm^3^ cm^-2^)	0.135 ± 0.008	0.124 ± 0.016	
LAR (cm^2^ g^-1^)	37.6 ± 1.6	38.2 ± 3.5	

*Significant influence of humidification (mixed model) is indicated by asterisks (P < 0.05). The influence of experimental plot was insignificant for all parameters. Live crown ratio, live crown length/tree height; M_stem_:M_fol_, ratio of stem to foliage dry weights; d^2^h:M_fol_, ratio of stem volume index to foliage dry weight; d^2^h:M_fol_, ratio of stem volume index to foliage area; LAR, leaf area ratio. N = 6 trees per treatment.*

**Table 4:** Biomass accumulation of birches in control (**C**) and humidified plots (**H**).

	C	H
Aboveground biomass (g m^-2^)	1001	862
Stems (g m^-2^)	518	437
Stem+Branches (g m^-2^)	800	691
Leaves (g m^-2^)	202	172
Leaves/BA (g m^-2^)	22.8	20.6
Leaf area index (m^2^ m^-2^)	3.76	3.13
Fine roots (g m^-2^)	93 ± 7	78 ± 14
Fine roots/BA (g m^-2^)	9.7	8.7
Fine root mass/leaf mass	0.46	0.45

*Aboveground biomasses and tree foliage area were calculated for every tree in the sample plots according to the linear regression models from d^2^h (**Table 2**) and expressed per stand area (m^-2^). All aboveground biomasses are linear transformations of d^2^h, and thus their response to humidification treatment is identical to the response of d^2^h (**Table 1**).*

### Effects on Leaf Characteristics

**FIGURE 1:**
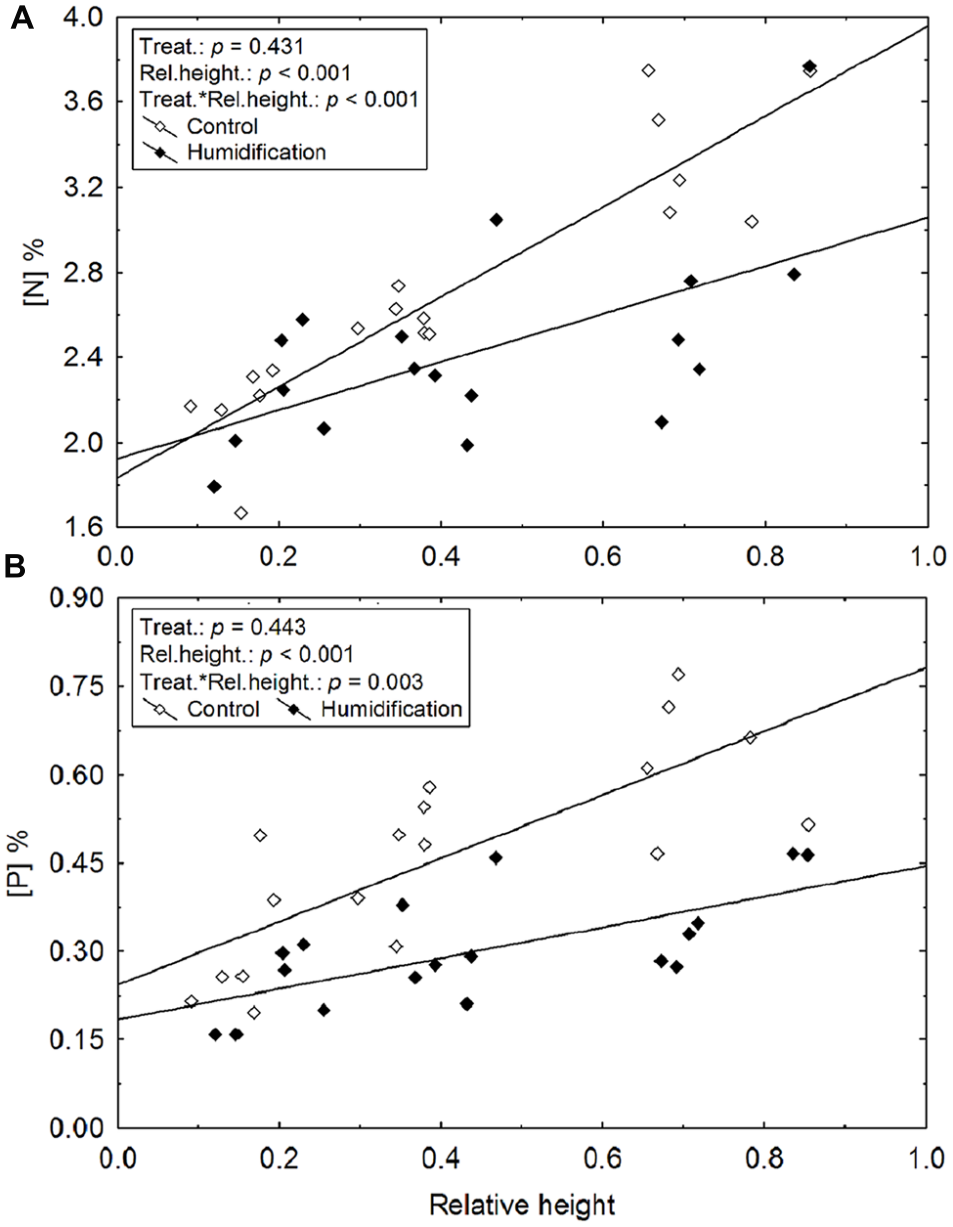
**The increase of leaf nitrogen (A) and phosphorus concentration (B) with relative height of branches (branch height/tree height) in control and humidified plots**.

Leaf nutrient concentrations were significantly affected by relative height of model branch (branch height/tree height) and by air humidification (**Figure 1**; **Table 5**). The experimental manipulation decreased leaf N and P concentrations (denoted as [N] and [P], respectively) in the upper crown (**Table 5**). [N] and [P] increased up the tree crown, while the slope of increase was smaller in humidified trees (**Figure 1**).

**Table 5:** Means of leaf parameters in three crown positions.

	Upper branch		Middle branch		Lower branch	
	C	H	c	H	C	H
[N] (%)	3.39 ± 0.13	2.70 ± 0.24^∗^	2.58 ± 0.04	2.40 ± 0.15	2.14 ± 0.10	2.19 ± 0.12
[P] (%)	0.624 ± 0.05	0.360 ± 0.03^∗∗^	0.468 ± 0.04	0.312 ± 0.04	0.302 ± 0.05	0.232 ± 0.03
N:P	5.67 ± 0.62	7.61 ± 0.46	5.80 ± 0.62	7.97 ± 0.50	7.86 ± 1.11	9.90 ± 0.76
N_area_ (g m^-2^)	1.95 ± 0.04	1.59 ± 0.17^∗^	1.50 ± 0.03	1.26 ± 0.07	1.15 ± 0.03	1.04 ± 0.07

*[N], leaf nitrogen concentration; [P], leaf phosphorus concentration; N:P ratio, and N_area_, leaf nitrogen content per unit leaf area. The significant effect of humidification on leaf parameters of a crown position (mixed model) is indicated by asterisks (^∗^P < 0.05, ^∗∗^P < 0.01). In a crown position, N = 6 trees per treatment.*

### Variation in Hydraulic Traits

**FIGURE 2:**
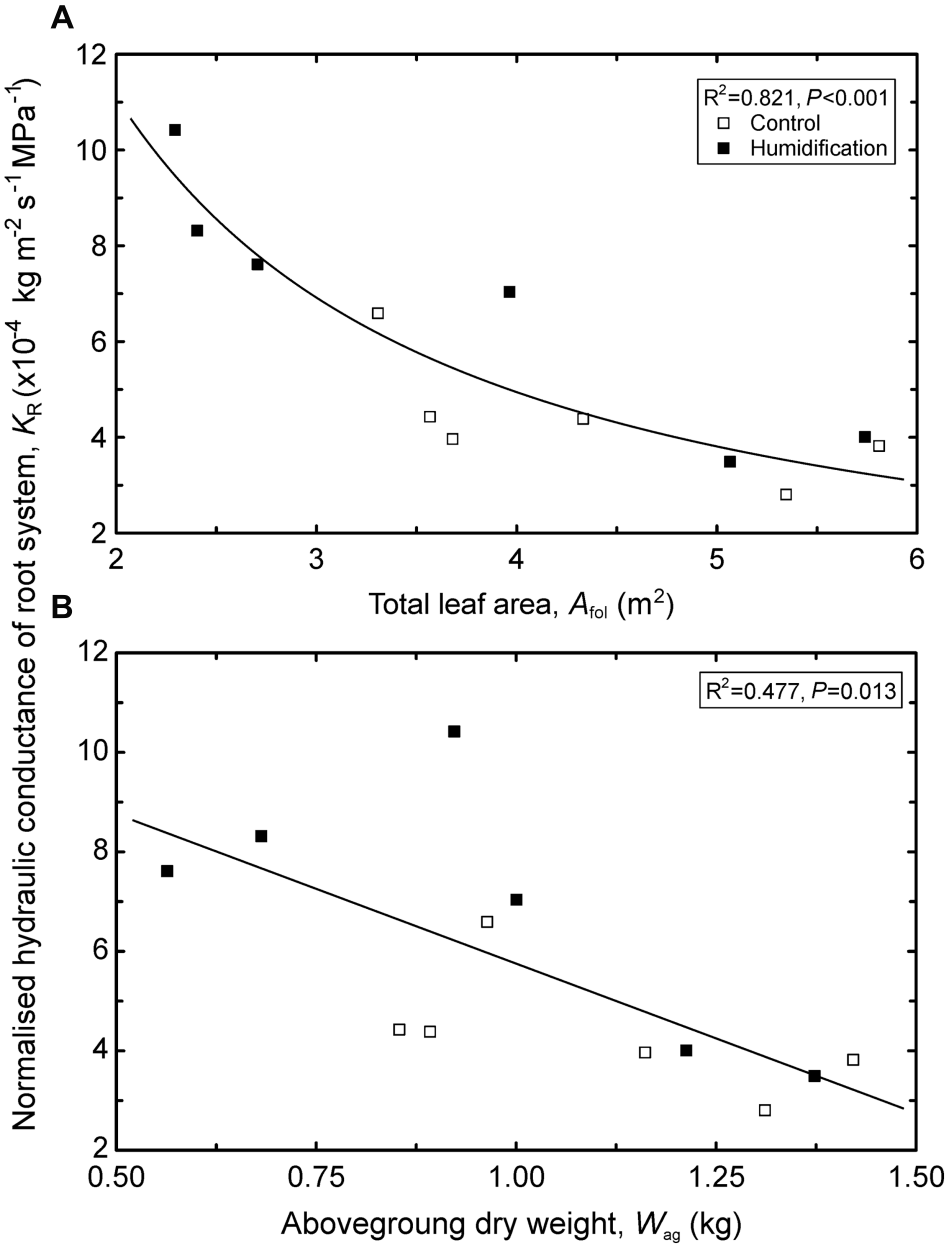
**Variation in normalized hydraulic conductance of the root system (*K*_R_) depending on tree total leaf area (*A*_fol_; A) and aboveground biomass (*W*_ag_; B).** Regression equations: **A** – y = 25.0x^-1.17^; **B** – y = -6.15 ⋅ 10^-4^x + 1.19 ⋅ 10^-4^.

The analysis of the hydraulic properties representing estimates for the whole crown or stem (i.e., means weighted by corresponding leaf areas) revealed that most of the traits did not significantly differ between control and humidified trees (**Table 6**). Although mean stem HV increased by 21% in response to elevated RH, the humidification effect was statistically insignificant. The *K*_Rabs_ depended neither on any tree’s size-related variable nor on the humidification treatment. In contrast, *K*_R_ changed with the experimental manipulation regardless of the trends in tree size: mean *K*_R_ increased from 4.33 × 10^-4^ kg m^-2^ s^-1^ MPa^-1^ in **C** plots to 6.85 × 10^-4^ kg m^-2^ s^-1^ MPa^-1^ in **H** plots. At the same time, *K*_R_ declined with increasing *h* (*R*^2^ = 0.360, *P* = 0.039), live crown length (*R*^2^ = 0.432, *P* = 0.020), *A*_fol_ (*R*^2^ = 0.821, *P* < 0.001; **Figure 2A**), and aboveground biomass (*R*^2^ = 0.477, *P* = 0.013; **Figure 2B**).

**Table 6:** Mean values of plant hydraulic traits in control and humidification treatments and treatment effect estimated with analysis of variance.

Trait	Control	Humidification	Treatment effect
Stem Huber value, HV (m^2^ m^-2^)	1.97 × 10^-4^	2.38 × 10^-4^	ns
Hydraulic conductance of root system, *K*_R_ (kg m^-2^ s^-1^ MPa^-1^)	4.33 × 10^-4^	6.85 × 10^-4^	*P* = 0.014
Absolute hydraulic conductance of root system, *K*_Rabs_ (kg s^-1^ MPa^-1^)	1.81 × 10^-3^	2.23 × 10^-3^	ns
Hydraulic conductance of leaves, *K*_L_ (kg m^-2^ s^-1^ MPa^-1^)	3.53 × 10^-4^	2.71 × 10^-4^	ns
Total hydraulic conductance of foliage, *K*_fol_ (kg s^-1^ MPa^-1^)	15.75 × 10^-4^	9.91 × 10^-4^	ns
*K*_Rabs_: *K*_fol_	1.29	2.55	ns
Relative branch hydraulic resistance, *R*_B_	0.470	0.385	*P* = 0.041
Relative leaf hydraulic resistance, *R*_L_	0.530	0.615	*P* = 0.041

*N = 6 trees per treatment; ns, not significant.*

**FIGURE 3:**
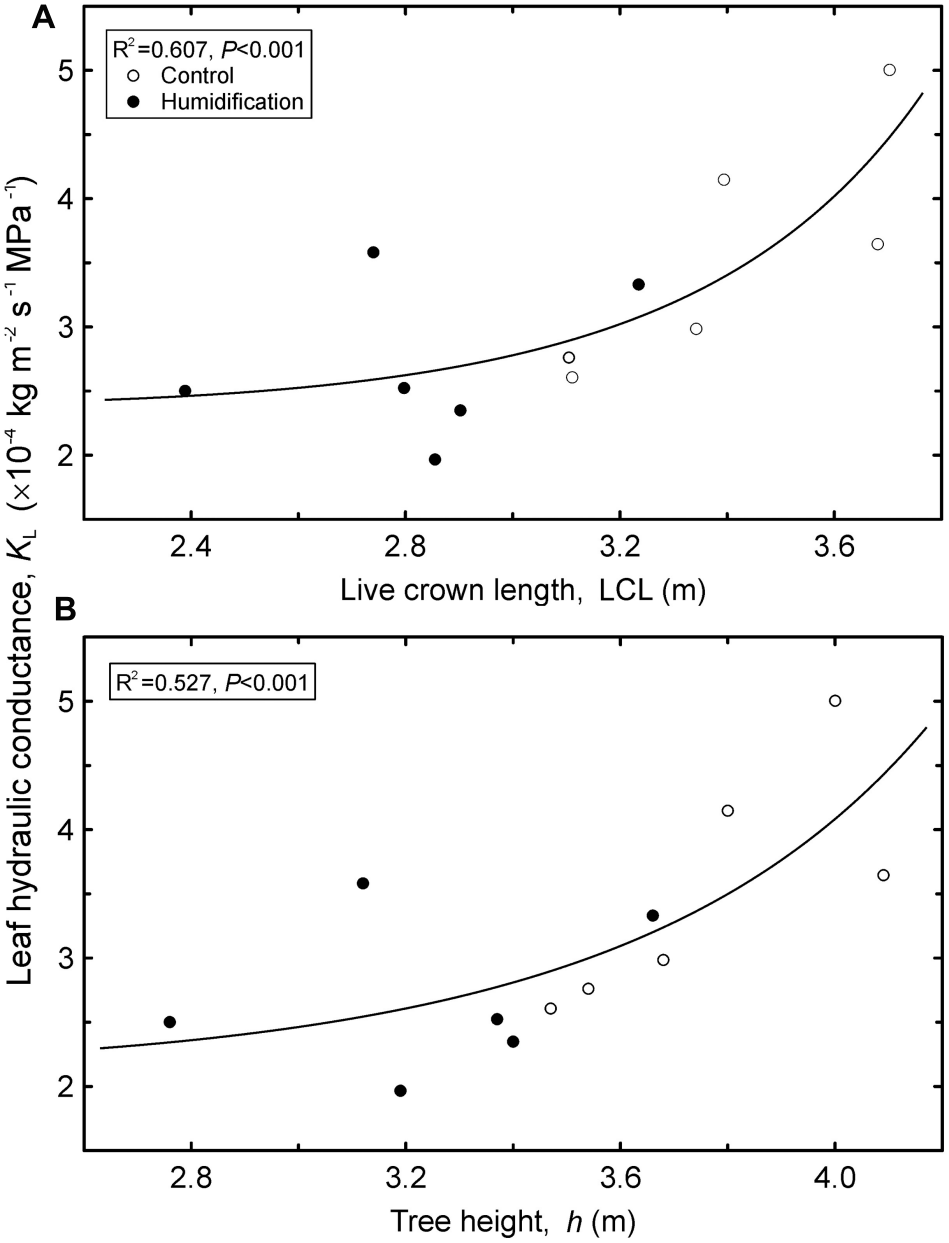
**Leaf hydraulic conductance (*K*_L_) calculated as a tree’s mean weighted by foliar area of the sample branches versus live crown length (LCL; A) and tree height (*h*; B).** Regression equations: **A** – y = 2.38 ⋅ e^1.04⋅10^-4^x^6.66^^; **B** – y = 2.15 ⋅ e^3.87⋅10^-4^x^5.35^^.

Although we recorded substantial decreases both in mean *K*_L_ and *K*_fol_, the treatment effects resulted in being statistically insignificant (**Table 6**). The *K*_fol_ increased with increasing tree aboveground dimensions, including *d*^2^*h* (*R*^2^ = 0.73, *P* < 0.001), live crown length (*R*^2^ = 0.762, *P* < 0.001), *A*_fol_ (*R*^2^ = 0.722, *P* < 0.001), and *h* (*R*^2^ = 0.684, *P* < 0.001). Tree *K*_L_, calculated as a mean weighted by foliar area of the sample branches taken from different crown layers, also increased with tree size (**Figure 3**) – with live crown length, *h*, and *d*^2^*h* (*R*^2^ = 0.62, *P* = 0.01). It is notable that mean tree *K*_L_ was independent of *A*_fol_ (*P* = 0.334). The increasing trend in root hydraulic conductance and decreasing trend in leaf hydraulic conductance induced by air humidity manipulation translated to an approximately two-fold increase in the *K*_Rabs_:*K*_fol_ ratio in **H** trees compared to control trees. Despite the substantial shift, ANOVA did not confirm significant impact of the treatment due to the dispersal of data among sample plots and separate sample trees.

**FIGURE 4:**
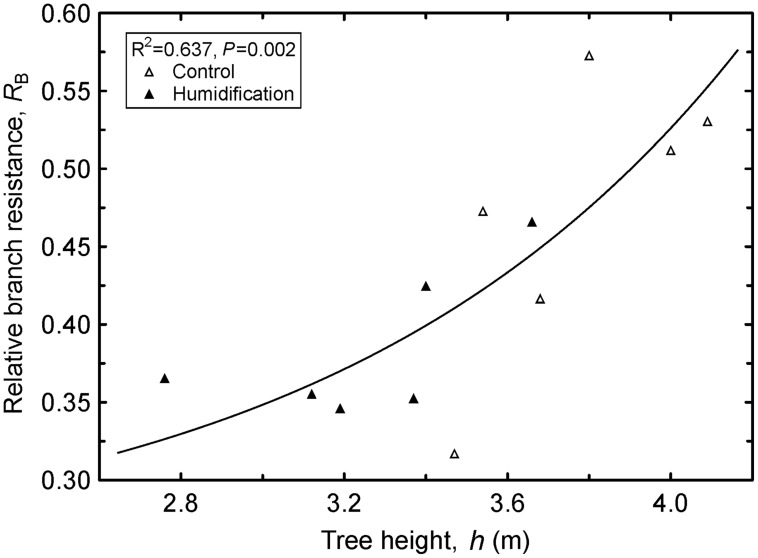
**Increase in relative branch hydraulic resistance (*R*_B_) with increasing tree height (*h*).** Regression equation: y = 0.276 ⋅ e^5.32⋅10^-3^x^3.46^^.

The humidity manipulation induced considerable shifts in the distribution of hydraulic resistance at the branch level; *R*_B_ decreased from 0.470 in **C** plants to 0.385 in **H** plants. Correspondingly, leaf contribution (*R*_L_) to branch total resistance changed in the opposite direction. The *R*_B_ increased (**Figure 4**) and *R*_L_ (*R*^2^ = 0.638, *P* = 0.002) decreased with tree height.

## Discussion

### Humidification Effect on Biomass

Exposure to increased relative air humidity decreased the aboveground biomass accumulation in saplings of silver birch (**Table 4**). As the shoots are under direct influence of atmospheric conditions, processes taking place in leaves and within-tree water transfer should be affected the most. Indeed, former studies conducted at the FAHM site revealed that humidification decreased transpirational flux, which, in turn, diminished leaf nutrient supply and photosynthetic capacity in humidified birches (Kupper et al., 2011; Sellin et al., 2013). The hampered mineral nutrition and photosynthesis result in a decrease of biomass production, as the current study demonstrates.

Humidified birches invested more resources to stem radial increment relative to height growth, likely to compensate for the lower mechanical strength of their stems due to reduced stem–wood density (**Table 3**). This is consistent with Christensen-Dalsgaard and Ennos (2012), who demonstrated decreased stem–wood strength of silver birch in wet conditions by comparing seedlings in well-watered conditions to those experiencing cyclical droughts. Also, Butler et al. (2012) indicated that woody plants with low stem specific gravity (defined as dry mass per fresh volume relative to density of water) have thicker stems. Since stem–wood density did not depend on aboveground biomass distribution (fraction proportions), the second hypothesis - the reduction of wood density of trees grown under high RH is associated with biomass distribution - remained unproven. Nevertheless, the decrease in stem density can be explained by mechanical load imposed on the stem, since **H** trees had relatively shorter crowns and smaller foliage (**Table 3**). The variation in mean branch-wood density (Sellin et al., 2013) is also attributable to the mechanical load subjected to the branch axis and is related to branch-level resource allocation (relatively larger foliar biomass results in denser branch-wood in control trees). The anatomical basis behind the shift in wood density is a subject of further studies. Christensen-Dalsgaard and Ennos (2012) did not find a relationship between stem–wood density and stem mechanical strength in silver birch, but this discrepancy can be explained by the very small size of 1-year-old seedlings used in their experiment.

Decreased height growth and the tendency of bigger branch proportions under humidification were contributed to also by desiccation of the top shoots, occurring more frequently in **H** plots. The top-shoot damage was induced by a fungal disease caused by *Cryptosporella betulae* (Tul. and C. Tul.) L.C. Mejía and Castl. (Hanso and Drenkhan, 2010). The top shoots died in ∼30% of humidified birches and only in 2% of untreated birches. In addition to impeded height growth, the death of top shoots usually enhances the growth of lateral shoots and, thus, increases branch proportion in the aboveground biomass. Certainly, the top-shoot damage was not the primary reason for the height growth suppression of the humidity-treated trees. Even the tallest uninfected trees in **H** plots remained shorter than the highest control trees; according to the frequency distribution of tree heights, the tallest height class was empty in **H** plots.

### Leaf Acclimations

Leaf [N] and [P] were lower under the humidification treatment (**Table 5**) as a result of decreased transpirational flux (Kupper et al., 2011), while an especially considerable decrease appeared in [P]. Unlike N nutrition, only a small (1–5%) amount of plant P demand comes via mass flow (Lambers and Plaxton, 2015), and plant P uptake depends mainly on functioning and properties of the roots. Since photosynthetic capacity of silver birch declines under elevated RH (Sellin et al., 2013), less photosynthates supporting P acquisition likely can be spent for roots and rhizosphere microorganisms in **H** plots, resulting in diminished nutrient acquisition, especially P uptake. An additional factor worsening leaf nutrient supply under increased atmospheric humidity is greater belowground competition, because root biomass of understory vegetation is more than two-fold higher in **H** plots compared to the controls (Kukumägi et al., 2014).

In addition to diminished nutrient uptake, probably also smaller foliage proportion in aboveground biomass (although statistically insignificant change) may restrict whole-tree photosynthesis and, thus, birch biomass accumulation in H plots, since biomass of photosynthesizing tissues is linearly related to plant-level photosynthetic productivity (Poorter et al., 2009). The retardation of foliage development seems to be a common response to elevated air humidity also observed in hybrid aspen in the FAHM experiment (Tullus et al., 2012). In humidified plots, where mineral nutrition and photosynthesis are impeded, SLA increased (Sellin et al., 2013) – it is an expected acclimation to enhance the leaf functional efficiency (efficiency of carbon sequestration or producing organic matter expressed per leaf area or mass unit) and assure tree growth. Thin leaves are cheaper to construct, as greater assimilating surface can be built per unit leaf dry weight. The increase in SLA of birch saplings with increasing RH is an expectable finding; it is a common plant response to humid environments (Niinemets, 2001; Warren et al., 2005). Despite higher SLA, LAI was lower under humidification, due to smaller tree size and foliar biomass fraction in the aboveground biomass (**Tables 3** and **4**). The LAR was not affected by humidification, because *A*_fol_ and aboveground biomass decreased to the same extent in response to elevated RH.

### Changes in Proportions of Non-photosynthetic versus Photosynthetic Tissues

Both stand-level leaf and fine-root biomasses were lower (15 and 16%, respectively) under humidification due to smaller tree size (**Table 4**). Similarly to SLA, the specific area of nutrient-absorbing ectomycorrhizal (EcM) root tips also increased (from 99 to 132 m^2^ kg^-1^) under humidification (Parts et al., 2013). Our results suggest that the initial acclimation response of *B. pendula* to increasing air humidity is the adjustment of plant morphology at the organ level rather than alteration of biomass distribution between the foliage and root system. Decreased nutritional status of humidified trees compared to controls induced morphological changes in EcM short roots (Parts et al., 2013), which are symptomatic for nutrient deficiency (Rosenvald et al., 2011; Ostonen et al., 2013), although the experimental plantation was established on fertile abandoned arable land.

The present study indicates that changes in HVs averaged across the entire stem resulted in being statistically insignificant (**Table 4**). The ratios of stem volume index to foliage dry weight or foliage area did not vary between the treatments, confirming that growing at elevated air humidity does not change resource allocation between non-photosynthetic and photosynthetic tissues in silver birch. Thus, the growth retardation cannot be associated with increasing proportion of sapwood leading to higher maintenance respiration costs (Ryan, 1990; Carey et al., 1997; Maier et al., 1998). The first hypothesis concerning changes in the ratio of non-photosynthetic to photosynthetic tissues was not confirmed. This contrasts with the data obtained on coppice of hybrid aspen that showed the stem-volume-to-leaf-area ratio was 15% greater (*P* = 0.033) in humidity-treated trees. Also, increased respiration costs might be the case if elevated RH expands a proportion of parenchymatous tissue in relation to dead xylem cells in sapwood. The anatomical analysis of hybrid aspen wood samples revealed a 5% greater proportion of pith rays in cross-sections of stems grown in **H** plots compared to the controls (Tullus et al., 2014). This issue remains to be elucidated by further anatomical analysis of birch stem wood.

### Changes in Hydraulic Capacity of the Root System

The increase of *K*_R_ in response to elevated air humidity is an unexpected result, because the average diurnal stem sap flux density per unit projected leaf area in silver birch trees was 24.8% (*P* < 0.05) and 27.2% (*P* < 0.01) higher in **C** plots compared to **H** plots during misting in 2008 and 2009 (Kupper et al., 2011), respectively, and there is no pressure for development of effective water uptake and transport system under reduced atmospheric evaporative demand. The ∼58% increase in *K*_R_ observed in humidified trees is primarily attributable to morphological modification of fine roots induced by the manipulation; trees grown under elevated RH exhibited smaller EcM short-root diameter and greater specific root length (SRL), specific root area, and short-root length (Parts et al., 2013). Steudle and Frensch (1996) indicated that the conductance of the root radial pathway is inversely proportional to the length of the flow path or the number of cell layers; therefore, smaller root diameter favors higher radial conductance. In *Citrus* species, the SRL of whole root systems is positively correlated with root hydraulic conductivity, while differences in overall root hydraulic conductivity among the citrus rootstocks are mainly related to structural differences in the radial pathway for water movement (Huang and Eissenstat, 2000). Consecutive studies (Pemán et al., 2006; Hernández et al., 2010) in different woody species confirmed the previous findings: species with thinner roots and great SRL exhibit high hydraulic conductance expressed per leaf surface area or stem cross-sectional area. Thus, the diminished nutrient uptake caused by reduced transpirational flux through the plants provokes root morphological alterations accompanied by increased hydraulic capacity. In addition, one cannot rule out effects resulting from changes in the ectomycorrhizal fungal community (Parts et al., 2013), as Xu et al. (2015) provide in their recent paper unequivocal evidence for the importance of fungal aquaporins in root water-transport efficiency of mycorrhizal plants.

Although neither surface area nor biomass of root systems were measured in the present study, but considering that below- and aboveground biomasses are tightly correlated, our data suggest that hydraulic efficiency of the root system declines with increasing tree size (**Figure 2**). The hydraulic limitation hypothesis proposed by Ryan and Yoder (1997) presents a mechanism explaining the deceleration of height growth with tree size and the maximum limits to tree height. The growth limitation of taller trees is primarily explained by growing resistance with increasing length of the hydraulic path and rising gravitational potential opposing the ascent of water in tall trees as well as with the growing ratio of non-photosynthetic to photosynthetic tissues (McDowell et al., 2002; Ewers et al., 2005; Ryan et al., 2006; Drake et al., 2010, 2011). The present results imply a novel aspect of the hydraulic limitation hypothesis: decreasing hydraulic efficiency of the root system with increasing tree dimensions. However, this issue needs verification in other species and plants covering a wider size range. The tree size-driven trend in *K*_R_ recorded in the present study coincides with that observed in seedlings of *Picea glauca* Moench Voss.: root hydraulic conductivity declines with an increasing root system size estimated by root surface area or dry weight (Krasowski and Caputa, 2005).

### Leaf versus Root Hydraulic Conductance

The air humidity manipulation reduced hydraulic conductance of leaf blades measured on separate sample branches by 19% (Sellin et al., 2013), but we did not establish a significant effect on *K*_fol_, the estimate of hydraulic efficiency of the entire foliage. This discrepancy can be explained by two matters. First, *K*_fol_ is based on whole-leaf measurements, embracing both petioles and lamina, while changes in petiole hydraulic conductance were statistically insignificant (see Table 5 in Sellin et al., 2013). Second, all whole-crown estimates of hydraulic properties represent weighted averages, in which the weight of lower canopy layers is remarkably larger than that of the upper canopy. However, RH deep inside the canopy is always higher and the humidification effect, therefore, less pronounced.

Elevated RH has a negative impact on both leaf vascular and extravascular pathways (Sellin and Alber, 2013). The approximate contribution of separate compartments to the decline of *K*_L_ in *B. pendula* was 14% for the petiole, 66% for the lamina vascular network, and 20% for the extravascular compartment. The strong positive relationships between *K*_fol_ and a range of parameters characterizing tree size constitute logical results. To provide adequate water delivery to mesophyll cells, the hydraulic capacity of shoots and their components must increase with increasing plant size. Taller trees with longer crowns have larger transpiring area, translated to a larger numbers of leaves connected in parallel (i.e., larger total cross-sectional area of vascular bundles in petioles and leaf veins), resulting in lower hydrodynamic resistance of the foliage. Our finding is consistent with the positive relationship between plant size and water transport capacity, a universal principle observed in single tree species as well as across species (Maherali and DeLucia, 2000; Tyree and Zimmermann, 2002; Lusk et al., 2007; McCulloh et al., 2014). Our earlier study also revealed linear relationships between total shoot hydraulic conductance and size characteristics in *B. pendula* (Sellin et al., 2012).

However, *K*_L_, calculated as a weighted mean across the whole crown, increased with tree height and crown length (**Figure 3**). Thus, the hydraulic efficiency of foliage expressed per leaf area unit increases with increasing tree height. In even-aged stands, this is attributable to increasing proportion of well-illuminated foliage with growing tree size; the *K*_L_ of sunlit leaves is 1.3–1.4 times higher than that of shade-grown leaves in silver birch, depending on particular conditions (Sellin et al., 2008, 2011; Õunapuu and Sellin, 2013). Well-exposed, higher-canopy leaves that face higher radiation load, temperature, and wind speed experience greater transpirational water loss. High atmospheric evaporative demand impels development of efficient water transport pathways of sun leaves to support higher transpiration rates.

Growing under higher atmospheric humidity caused *K*_R_ to increase and *K*_L_, rather, to decrease in saplings of silver birch (Sellin et al., 2013), which translated to considerable increase in *K*_Rabs_:*K*_fol_ values (**Table 6**). Although the treatment effect was statistically not confirmed due to high variation among sample plots, this shift implies probable alteration in distribution of liquid-phase resistances at the whole-tree level. However, the efficiency of the water transport system is coordinated at different points in the entire transport pathway from roots to leaves. Thus, higher stem hydraulic efficiency is associated with higher root and leaf hydraulic efficiencies (Pratt et al., 2010). Under stable environmental conditions, the changes observed in silver birch likely do not affect normal tree functioning, but they could play a crucial role under severe stress conditions (heat wave or strong drought) as we can suppose based on the previous results from the FAHM experiment (Niglas et al., 2014; Sellin et al., 2014). Larger hydraulic conductivity of roots is usually accompanied by increased susceptibility to cavitation (Domec et al., 2009), and this may limit leaf gas exchange in stress conditions. Therefore, canopy conductance of hybrid aspen decreased 3.2 times faster in response to falling soil water potential in the **H** treatment compared to the controls (Niglas et al., 2014). Furthermore, reduced hydraulic efficiency of leaves and disproportionate changes in sensitivity of stomatal conductance versus *K*_L_ to water deficit (Sellin et al., 2014) during sudden weather fluctuations will impose greater risk of desiccation-induced hydraulic dysfunction on plants developed under high atmospheric humidity and could represent a potential threat to hemiboreal forest ecosystems. The crucial role of leaf liquid-phase conductance in whole soil-to-leaf water transport pathways in rapidly changing conditions is also noticeable at the branch level: *R*_L_ increased from 53.0 to 61.5% in response to elevated air humidity (**Table 4**). This is attributable to both decreased *K*_L_ and a decreased proportion of well-illuminated canopy due to shorter trees in humidified plots (Sellin et al., 2013). Thus, both the trends in *K*_R_ and *K*_Rabs_:*K*_fol_ induced by elevated atmospheric humidity attest to the significant role of tree size in their acclimatory responses, supporting the third hypothesis.

## Conclusion

Two years of application of increased air humidity decreased the growth rate and biomass accumulation in saplings of *B. pendula*. The humidity manipulation affected plant morphology (live crown length, slenderness, SLA, and fine-root traits) and wood density rather than biomass distribution among aboveground organs. The pronounced changes occurred in morphological and functional characteristics of leaves and fine roots (i.e., in plant parts consisting mainly of living tissues and implementing the most basic physiological processes, such as assimilation of CO_2_ and nutrient and water uptake). The treatment had a significant effect on several hydraulic properties of the trees, while the shifts were largely associated with changes in tree size but not in biomass allocation. Our results prove a sensitivity of tree biomass accumulation and hydraulic architecture to regional climate trends (i.e., increasing atmospheric humidity) predicted for northern Europe.

## Conflict of Interest Statement

The authors declare that the research was conducted in the absence of any commercial or financial relationships that could be construed as a potential conflict of interest.
